# Geostatistical Mapping of Salinity Conditioned on Borehole Logs, Montebello Oil Field, California

**DOI:** 10.1111/gwat.13155

**Published:** 2021-12-16

**Authors:** Neil Terry, Frederick Day‐Lewis, Matthew K. Landon, Michael Land, Jennifer Stanton, John W. Lane

**Affiliations:** ^1^ Pacific Northwest National Laboratory Earth Systems Science Division, EED Richland WA USA; ^2^ U.S. Geological Survey, California Water Science Center Sacramento CA USA; ^3^ U.S. Geological Survey, New England Water Science Center Reston VA USA; ^4^ U.S. Geological Survey, Earth System Processes Division, Hydrogeophysics Branch Storrs CT USA

## Abstract

We present a geostatistics‐based stochastic salinity estimation framework for the Montebello Oil Field that capitalizes on available total dissolved solids (TDS) data from groundwater samples as well as electrical resistivity (ER) data from borehole logging. Data from TDS samples (*n* = 4924) was coded into an indicator framework based on falling below four selected thresholds (500, 1000, 3000, and 10,000 mg/L). Collocated TDS‐ER data from the surrounding groundwater basin were then employed to produce a kernel density estimator to establish conditional probabilities for ER data (*n* = 8 boreholes) falling below the selected TDS thresholds within the Montebello Oil Field area. Directional variograms were estimated from these indicator coded data, and 500 TDS realizations from conditional indicator simulation were generated for the subsurface region above the Montebello Oil Field reservoir. Simulations were summarized as 3D maps of median TDS, most likely salinity class, and probability for exceeding each of the specified TDS thresholds. Results suggested TDS was below 500 mg/L in most of the study area, with a trend toward higher values (500 to 1000 mg/L) to the southwest; consistent with the average regional groundwater flow direction. Discrete localized zones of TDS greater than 1000 mg/L were observed, with one of these zones in the greater than 10,000 mg/L range; however, these areas were not prevalent. The probabilistic approach used here is adaptable and is readily modified to include additional data and types and can be employed in time‐lapse salinity modeling through Bayesian updating.

## Abstract 2

*Article impact statement*: A three‐dimensional adaptable geostatistical approach classifying groundwater salinity above the Montebello oil field is developed and applied.

## Introduction

Los Angeles County in Southern California is a densely populated region with more than 900 residents per km^2^ (U.S. Census Bureau 2018), has a heavy reliance on groundwater as a water supply (Reichard et al. 2003; Edwards et al. 2009), and is an area of historical and continuing oil and gas development (Gautier et al. 2012). Development of oil and gas wells began in Los Angeles in the late 1800s, and as of 2018 Los Angeles County included more than 4400 active and more than 15,000 plugged and abandoned oil and gas wells (California Department of Conservation 1992, 2018a). The relative proximity of groundwater resources to intensive oil and gas development and the high volume of subsurface fluid injection led to several areas of the Los Angeles Basin being classified as high priority for regional monitoring of groundwater quality (Davis et al. 2018; U.S. Geological Survey 2019).

A program to monitor water quality in areas of oil and gas development was authorized under California Senate Bill 4 of 2013 (SB4; State of California 2013) and implemented by the California State Water Resources Control Board (2018a). The U.S. Geological Survey (USGS) is collaborating with the California State Water Resources Control Board to conduct regional groundwater monitoring that includes assessing: (1) the location and characteristics of potentially usable groundwater resources in proximity to oil and gas operations; (2) evidence of fluids from oil and gas sources in groundwater, and, if present, pathways or processes that may be responsible; and (3) how oil and gas operations have affected groundwater quality relative to other processes (USGS 2019).

Historically, both the California State Water Resources Control Board and the California Department of Conservation, Geologic Energy Management Division (CALGEM) have defined total dissolved solids (TDS) thresholds for groundwater resources considered for specific restriction from oil and gas activities as those containing less than 3000 mg/L TDS (Horsley Witten Group 2011). However, more recently the State has adopted the U.S. Environmental Protection Agency definition of underground sources of drinking water as nonexempt aquifers having less than or equal to 10,000 mg/L TDS (California State Water Resources Control Board 2015a). The nonregulatory, aesthetic‐based secondary maximum contaminant level range for drinking water is 500 to 1000 mg/L TDS (California State Water Resources Control Board 2015b). Therefore, TDS values of 500, 1000, 3000, and 10,000 mg/L serve as notable thresholds of relevance for mapping subsurface salinity near oil fields (Stanton et al. 2017). In this paper, we have adopted terminology set by the National Ground Water Association (NGWA 2013) to describe categories for groundwater salinity based on TDS: less than 1000 mg/L (fresh), from 1000 to 3000 mg/L (slightly saline), from 3000 to 10,000 mg/L (moderately saline), and greater than 10,000 mg/L (highly saline).

Directly sampled TDS data provides critical information for groundwater salinity mapping. However, the cost and labor required to install and maintain wells is limited to select areas. Therefore, secondary sources of information related to TDS, as well as methods for combining various information types, are needed to upscale directly measured TDS data as part of the regional salinity mapping effort near oil fields (Gillespie et al. 2017, 2019; Stephens et al. 2019; Ball et al. 2020). For example, groundwater electrical conductivity is closely related to TDS (being a measure of total ions in solution) and can therefore be used to indirectly estimate TDS. Bulk electrical resistivity (ER) measurements are sensitive to variations in groundwater electrical conductivity and therefore TDS as well, though ER measurements are also influenced by soil/rock properties and temperature. An abundance of historical ER information for the area exists in the form of archived oil well borehole geophysical logs (California Department of Conservation 2018b). While numerous studies have used borehole geophysical log data to estimate groundwater salinity (e.g., Archie 1942; Jorgensen 1989; Keys 1990; Wonik and Olea 2007), geostatistical methods provide an opportunity to incorporate uncertainties in the various data types and spatial structure to map salinity at broader scales.

There is extensive information to support developing relations between ER data from borehole geophysical logs and TDS in the Los Angeles Basin. The Water Replenishment District of Southern California (WRD), working in cooperation with the USGS, has a groundwater network including 59 multiple well monitoring sites with 322 monitoring wells at different depths in the Central and West Coast Basins of Los Angeles County; these sites include aquifer lithology and properties, borehole geophysical logs, and water chemistry data (Land et al. 2002; Reichard et al. 2003; Water Replenishment District of Southern California 2018, 2020a, 2020b). TDS measured in these wells and water‐supply wells and limited produced water samples from oil wells (Metzger et al. 2018, 2020) have been used to inform the distribution and quality of groundwater resources. However, groundwater data are typically sparser in oil field areas than in other parts of the basin (Davis et al. 2018; Metzger and Landon 2018).

**Figure 1:**
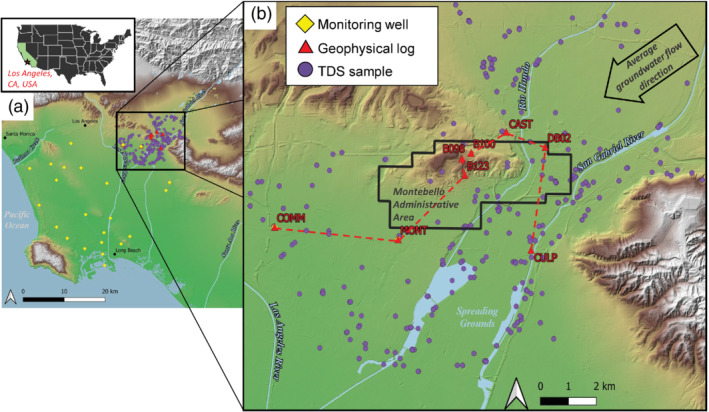
Location of the study area: (a) coastal plain of the Los Angeles Basin in Los Angeles County with locations of monitoring wells, borehole geophysical logs, and TDS samples shown and (b) detail of simulation area with location of transect between the geophysical log locations in the vicinity of the Montebello Oil Field and regional groundwater flow direction. Basemap data are from the U.S. Geological Survey.

In this study, we incorporated available TDS and ER data into a geostatistical indicator simulation framework as a pilot effort to refine methods used to map salinity overlying the Montebello Oil Field in the northeastern Los Angeles Basin (Figure 1). The spatial correlation structure of TDS was estimated via experimental variograms computed from the relatively large number of geophysical logging data (ER logs) available at the pilot study area, as well as TDS data measured from samples taken from monitoring wells. The ER data were then treated as “soft” indicator variables that were categorized as probabilities of falling below certain TDS thresholds of interest (500, 1000, 3000, 10,000 mg/L) based on a calibration with collocated ER‐TDS data. The directly measured “hard” TDS data and the ER‐based probabilities, combined with information from the variograms, were used to simulate equiprobable distributions of TDS. After 500 simulations, we estimated the most likely TDS distribution throughout the study area and probabilities for existing within each TDS class (fresh, slightly saline, moderately saline, and highly saline).

## Background

### Montebello Oil Field Study Area

The Montebello Oil Field area (Figure 1b) was selected as a pilot study area for developing geostatistical methods for salinity mapping for multiple reasons, including: (1) among approximately 500 onshore oil fields in California, the Montebello Oil Field was classified as a high‐priority area to consider implementing regional groundwater monitoring due to a relatively high volume of water injected into the field and a high density of petroleum wells (Davis et al. 2018); (2) it is proximal to major managed and natural regional recharge areas for the coastal plain of the Los Angeles Basin, providing water supply to millions of people; and (3) there are extensive water chemistry, borehole log, and supporting hydrogeologic framework data in this area (Ponti et al. 2014; Water Replenishment District of Southern California 2018).

The Los Angeles Basin formed during the late Miocene on a continental margin that had previously experienced Mesozoic and early Paleogene subduction, Paleogene terrane accretion, and mid‐Miocene rifting and block rotation (Wright 1991). Transtensional deformation (normal and strike‐slip faulting) and an eastward shift in the southern San Andreas Fault during the early Pliocene resulted in basin subsidence. During the middle Pliocene changes in relative plate motion induced north‐south shortening, propagation of blind thrusts beneath the basin, and basin filling that continues into the present. Pliocene‐ to Holocene‐age deposits in the Los Angeles Basin were characterized based on unconformities mapped from oil industry seismic data (Ponti et al. 2014).

**Figure 2:**
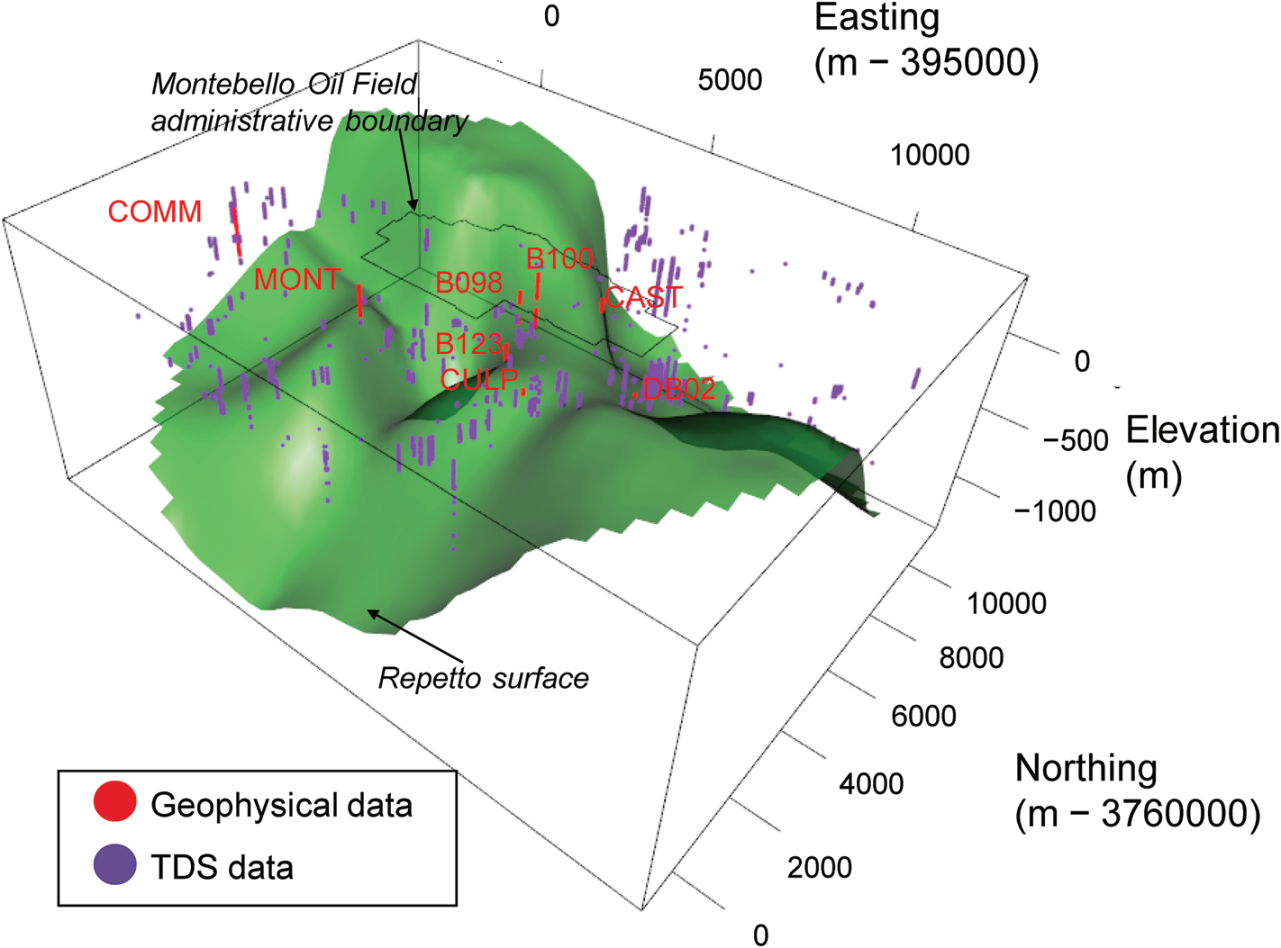
Three‐dimensional view of a digital elevation model of the top of the Repetto Formation in the Los Angeles Basin (United States) with positions of borehole geophysical logs and TDS data shown.

The Montebello Oil Field is in the Merced‐Puente Hills near the northeastern edge of the Los Angeles Basin (Reichard et al. 2003). A three‐dimensional chronostratigraphic layer model that classifies sediments into 14 chronostratigraphic layers has been developed for the Los Angeles Basin (Ponti et al. 2007, 2014), including 12 upper layers that are the primary water bearing units and the underlying Repetto Formation and Miocene layers that are the primary oil‐bearing units. In the Montebello Oil Field area, the sediments that are the main water‐bearing units are relatively thin, as little as 250‐m thick over the oil field, and deepen to greater than 1000 m downslope to the southwest and northeast (Figure 2). On average, the water‐bearing sediments are 560‐m thick in the study area.

The Montebello Oil Field was discovered in 1917 on the Anita Baldwin property by Standard Oil (California Department of Conservation 1992). Since then, 832 oil and gas wells have been drilled in the Montebello Oil Field, of which 228 are classified as active or inactive, and 604 are classified as plugged or buried (California Department of Conservation 2018a). There are eight pools developed for oil extraction in the Pliocene‐aged Repetto and Miocene‐aged Puente Formations (California Department of Conservation 1992). Oil wells in the Montebello Oil Field have depths to the top of perforations ranging from 160 to 2600 m, with a mean of about 1155 m. Enhanced oil recovery, primarily from injection of recycled produced water into oil‐bearing formations (water flooding), began in 1960 (California Department of Conservation 1992). There are currently 63 active or inactive water‐flood wells in the Montebello Oil Field (California Department of Conservation 2018a), with mean depths to the top of perforations of about 700 m. Most injection is into the upper two oil pools in the Repetto Formation in the main area of production (California Department of Conservation 1992). Approximately 105 million cubic meters of water were injected during 1956 to 2017 (California Department of Conservation 2018b, 2020).

The Montebello Oil Field is just north of the Rio Hondo and San Gabriel River spreading grounds (Figure 1b), which are managed aquifer recharge areas that are a major source of recharge to the regional groundwater flow system of the Central and West Coast groundwater basins that supplies water to the cities of the Los Angeles Basin (Reichard et al. 2003). The groundwater system directly adjacent to the Montebello Oil Field is heavily exploited for water supply; there are approximately 183 public supply wells located within 5 km of the administrative boundary of the Montebello Oil Field. However, groundwater development within the administrative boundary of the oilfield is relatively sparse (about 5 public‐supply wells) as the shallowest most permeable upper layers of the aquifer system are thin or absent overlying the uplifted area of the oil field, providing lower yields than adjacent alluvial valley deposits south and east of the field. Groundwater‐level elevations indicate that regional groundwater flows from the northeast to southwest along the axis of the oil field (Figure 1b) and off the oil field into surrounding heavily developed groundwater areas (Reichard et al. 2003; Water Replenishment District of Southern California 2018).

**Figure 3:**
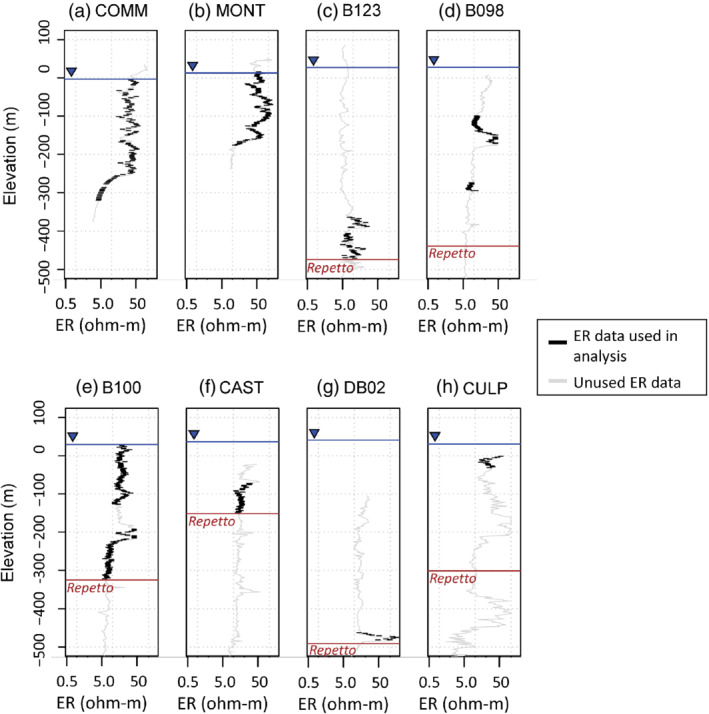
Electrical resistivity (ER) log data from 8 borehole geophysical logs versus elevation highlighting data that were used in the analysis (below water table, above Repetto Formation in the Los Angeles Basin, and corresponding to sandy units).

Borehole geophysical logs from the area of the Montebello Oil Field were used in this study, with locations shown in Figures 1b and 2, and ER values extracted from the borehole geophysical logs (Figure 3). Wells with associated TDS data also occur in this area (Figure 1b). The bottom of the domain in our analysis was the top of the Repetto Formation (Ponti et al. 2014, Figure 2); this layer was excluded because it is not a major water‐bearing unit and contains oil, which alters the apparent relations between borehole resistivity and calculated TDS.

### Stochastic Conditional Simulation

Geostatistical approaches are used to estimate properties that vary in space and/or time and include methods such as interpolation, kriging, and conditional simulation. While kriging minimizes estimation variance at unknown locations to produce a single final model (Olea 1991), simulation approaches instead produce many models, each of which honors the data and global covariance structure. Unlike kriging alone, by simulating many realizations, more detailed properties of the estimation uncertainty can be evaluated (Deutsch and Journel 1998).

Stochastic simulation operates by producing individual realizations (several hundred or more) that honor measured data and the specified model of spatial variability, for example, a variogram model (Srivastava 1992; Froidevaux 1993). Each realization is produced through a random walk through each grid point in a defined 2D or 3D simulation grid, where a kriging (or cokriging) system is solved to estimate the value at that point using the given variogram model, observed data, and all previously simulated points. In practice, the total number of data and simulated points used to condition the kriging system at each grid point is limited.

The distribution of TDS values simulated at each grid point can be used to estimate the most likely value at that point and assess the uncertainty in model prediction. By comparison to traditional kriging, uncertainty assessment from simulation is not limited to a summary statistic (i.e., the variance) that assumes a parametric distribution of estimated values at each point; an assumption that may not be appropriate in regions of complexity where data show extreme variation. Rather, results from simulations can be summarized as histograms to assess characteristics such as multimodality, as might occur in areas where water from a saline source mixes with a freshwater source. Furthermore, such probabilistic outputs can be used as inputs in subsequent Bayesian modeling frameworks.

### The COSISIM Algorithm

The combination of available data and objectives in this study was amenable to a stochastic conditional simulation approach using indicator‐coded variables, such as is implemented in the SGeMS COSISIM algorithm (Remy 2005). SGeMS is a graphical user interface to run a variety of geostatistical algorithms. COSISIM simulates models based on a global covariance structure and probabilistic data inputs that inform the likelihood of the study area falling into different parameter categories. In this case, we specifically wanted to know where groundwater salinity is most likely less than or equal to 500 mg/L, less than or equal to 1000 mg/L, less than or equal to 3000 mg/L, less than or equal to 10,000 mg/L, or greater, given directly measured TDS values and indirect ER logging information.

Therefore, we employed the following model framework for input of data into COSISIM (e.g., Deutsch and Journel 1998). TDS is an intrinsic property of water in sediments; a continuous variable denoted as the vector Z. Direct measurements of TDS exist in the study area at discrete locations and TDS is our primary variable of interest; thus, these measurements are considered “hard” information. Thresholds for groundwater salinity of regulatory significance include k=4 TDS cutoffs of 500, 1000, 3000, and 10,000 mg/L. We symbolize these cutoffs as z with $z_{1}$ = 500 mg/L, $z_{2}$ = 1000 mg/L, $z_{3}$ = 3000 mg/L, and $z_{4}$ = 10,000 mg/L. The TDS measurements, Z, can be coded as indicator variables for each cutoff (*z*). Indicator‐coded *Z* values are represented as $I^{\text{hard}}$. Rows of $I^{\text{hard}}$are datapoint locations, while the columns are four values corresponding to the four TDS thresholds (z), that is, the first row of $I^{\text{hard}}$ is $\left[{i^{\text{hard}}}_{1},{i^{\text{hard}}}_{2},{i^{\text{hard}}}_{3},{i^{\text{hard}}}_{4}\right]$, where ${i^{\text{hard}}}_{1}$, $\;{i^{\text{hard}}}_{2}$, ${i^{\text{hard}}}_{3}$, and ${i^{\text{hard}}}_{4}$ are equal to 1 if they are less than or equal to the threshold values, and 0 otherwise. For example, a TDS value of 3500 mg/L would be coded as [0,0,0,1].

ER informs TDS to some extent and is thus considered “soft” information. Here we refer to available soft information (electrical geophysical logs) as the vector Y. We possess calibration datasets outside the Montebello study area but within the larger coastal plain of the Los Angeles Basin (that includes Montebello) with collocated ER‐TDS information. Provided this calibration dataset covers the range of values expected to occur in the study area, as it does in this study, it can be used to provide a probabilistic estimate of TDS values occurring for different values of ER. Such a probabilistic estimate can be produced through a kernel density estimator using the calibration datasets (Holmes et al. 2007). Probabilities derived from the kernel density estimator can then be transformed to conditional cumulative distribution functions (ccdfs). The value of the ccdfs at the cutoffs ($z_{1}$,$\;z_{2}$,$\;z_{3}$, and $z_{4}$) provide an indicator coding for the soft data (Y) that can be used in simulation. In this case, the indicator coding is the probability that the soft data will be less than or equal to the four thresholds. The indicator‐coded soft data are referred to as $I^{\text{soft}}$. Similar to the hard data, for each soft datapoint, there are four probabilities for being less than or equal to the four TDS thresholds, that is, one row of $I^{\text{soft}}$ is represented as $\left[{i^{\text{soft}}}_{1},{i^{\text{soft}}}_{2},{i^{\text{soft}*}}_{3},{i^{\text{soft}}}_{4}\right]$ with each value of these values having a minimum of 0 and maximum of 1. For example, suppose, based on analysis of collocated TDS‐resistivity information or some other prior knowledge/model, a resistivity datapoint has a 0% chance of being less than or equal to 500 mg/L, a 25% chance of being less than or equal to 1000 mg/L, a 75% chance of being less than or equal to 3000 mg/L, and a 100% chance of being less than or equal to 10,000 mg/L. Such a datapoint would be coded as [0.00, 0.25, 0.75, 1.00].

Indicator simulations are performed in COSISIM by successively solving an indicator cokriging system for each point in a user‐defined simulation grid, where the simulation grid consists of a 2D or 3D discretization of the spatial area of interest and contains of a total of $n_{\text{grid}}$ cells. In our case, we simulated values in the 3D subsurface space between the water table and the top of the Repetto Formation. We therefore established a grid containing $n_{\text{grid}}$ = 504,300 cells (each cell was 100 × 100 × 25 m^3^).

We denote the matrix of simulated indicator values from COSISIM as $I^{\mathrm{est}}$, which is $n_{\text{grid}}\times k$ in size. Each simulation begins with a randomly determined starting grid point, wherein an indicator cokriging system is solved for that point using observed data and a given covariance model. The indicator cokriging estimates at the initial location are probabilities for each of the indicator cutoffs, and are thus represented as a discrete representation of a ccdf with similar format to the input indicator data $I^{\text{hard}}$ and $I^{\text{soft}}$. After estimating an initial value, the simulation follows a random walk through the entire simulation grid. Successive points use both observed data and previously simulated values to solve the cokriging system. In practice, the number of conditioning data used to solve the cokriging system (i.e., the $I^{\text{hard}}$ and $I^{\text{soft}}$ hard and soft data as well as previously simulated points) are limited by a maximum search radius and total number of points to consider. This limiting of the search neighborhood is necessary for computational efficiency.

After every point has been simulated and every row and column of $I^{\mathrm{est}}$ has been filled, results are back‐transformed within COSISM to original data units (i.e., TDS in mg/L), which we shall denote as the vector $Z^{\mathrm{est}}$ (and is of $n_{\text{grid}}$ length). This is accomplished through an interpolation (between TDS cutoff values where the ccdfs are defined) and tail extrapolation (i.e., extrapolating to a ccdf value of 0 between the global minimum data value and the first cutoff, and extrapolating to a ccdf value of 1 between the last cutoff and the maximum global data value) of the individual discretized ccdfs contained within $I^{\mathrm{est}}$ and sampling a value from those distributions. The global minimum and maximum data values were established as 60 and 14,000 mg/L TDS, respectively, based upon the TDS values within the study area and larger region.

Many ($n_{\mathrm{sim}}$) realizations of TDS values over the simulation grid are created through conditional simulation in COSISIM, and so the final result is an $n_{\text{grid}}\times n_{\mathrm{sim}}$ matrix, $Z^{\mathrm{sim}}$. From each row of $Z^{\mathrm{sim}}$, corresponding to all realizations for a particular voxel in the simulation grid, a histogram of the simulated TDS values can be produced to summarize results. The relative counts of TDS values in each histogram bin indicate the relative TDS probability. These histograms can be used to approximate probability density functions or derive summary statistics, such as median TDS values, most likely TDS class, or probabilities of exceeding TDS thresholds.

### Covariance Inference

To perform indicator simulations, it is necessary to repeatedly solve a cokriging system that predicts indicator‐coded TDS values based on $I^{\text{hard}}$ and $I^{\text{soft}}$ throughout the simulated study field. Solving for the weights in this cokriging system also technically requires a covariance function for each variable, in this case $I^{\text{hard}}$ and $I^{\text{soft}},$ as well as a cross‐covariance function between those variables for each indicator cutoff. Developing these functions (as from variograms) may prove unrealistic. For example, in our case we consider k=4 indicator cutoffs and two spatial correlation directions (lateral and vertical), creating a need for 16 covariance functions and 32 cross‐covariance functions. We can reduce the number of covariance matrices needed to one per direction by using the Markov‐Bayes algorithm (Zhu 1991; Zhu and Journel 1993) and a median indicator kriging assumption. The Markov‐Bayes algorithm is used to estimate parameters (the hardness criteria, see next paragraph) that obviate the need for separate covariance and cross‐covariance functions. Thus, only the covariance functions for the primary indicator variable are needed, which can be supplied by variogram models estimated from hard and/or soft data. If it can be assumed that the indicator variables for the different cutoffs are intrinsically correlated (Goovaerts 1997), it is further possible to use the same variogram model for each of the primary indicator variables. This simplification, referred to as median indicator kriging, is one we adopt here.

The Markov‐Bayes model employs a “hardness” criterion, B(z), for each indicator cutoff (z). Effectively, this is a gauge of the strength of the relationships between soft and hard data for each threshold and can be used as a proxy for soft data covariance and cross‐covariance functions. The hardness criteria, B(z) (vector of length k), describes how well soft data can be used to predict hard data classes with B(z) = 1 equating to a perfect prediction, and B(z) = 0 indicating no ability to use soft data to inform hard data for a class. A further discussion of the calculation and meaning of the B(z) values is discussed in the Supporting information, but the overall utility of this approach is that the B(z) values can be used within the solution of the cokriging system instead of providing separate covariance and cross‐covariance functions for the soft data ($I^{\text{soft}}$). Therefore, between employing this Markov‐Bayes model and making the median indicator kriging assumption, the need to estimate variograms is reduced to just one model per direction (lateral and vertical).

## Methods

### Data Preparation

Many oil wells have archived scanned (California Department of Conservation 2018b) or paper copies of borehole logs collected after drilling. Available scanned borehole geophysical logs within the study area were collected during 1918 to 2012 with a median date of 1949 and thus represent a wide range of borehole log types of varying quality. A subset of six oil well borehole geophysical logs (positions shown in Figure 1b, data shown in Figure 3) were selected based on having ER data of sufficient quality to be digitized and having information within the sandy water‐bearing units. Digitizing was done using a commercial software program from Neuralog (www.neuralog.com). Modern borehole geophysical log data are also collected with the installation of groundwater monitoring wells such as the Montebello 1 site (Land et al. 2002) located near the southwestern edge of the Montebello Oil Field (labeled “MONT” in Figures 1b, 2, and 3b). The Commerce 1 site (“COMM” in Figures 1b, 2a, and 3a) is another monitoring well with modern borehole geophysical log data that we use in our analysis and calibration of the dataset (see “calibration datasets” section below). Altogether, ER data from eight borehole geophysical logs were used in the simulation, including the MONT and COMM monitoring wells and the six borehole geophysical logs (Figures 1b, 3, Table 1). These data comprise the vector Y.

**Table 1:** Information Available from the Eight Borehole Geophysical Logs Used for the Geostatistical Estimation

Common Name (abbreviation)	Identifier (API or NWIS number)	Electrical Log Type Used	Other Information	Depth Range (m)	Collection Date
Commerce‐1 (COMM)	USGS^1^ 340040118100901	Long‐normal resistivity	Gamma, self‐potential, induction, short‐normal resistivity, TDS	12‐424	1999
Montebello‐1 (MONT)	USGS^1^ 340027118071901	Long‐normal resistivity	Gamma, self‐potential, induction, short‐normal resistivity, TDS	9‐298	2001
Baldwin 123 (B123)	03710841^2^	Lateral resistivity	None	40‐671	1944
Baldwin 98 (B098)	03710818^2^	Lateral resistivity	Self‐potential, short‐normal resistivity	152‐944	1943
Castle 1 (CAST)	03705372^2^	Lateral resistivity	Short‐normal resistivity	115‐1585	1951
Baldwin 100 (B100)	03710820^2^	Lateral resistivity	Self‐potential, short‐normal resistivity	107‐853	1943
Davis Baldwin 2 (DB02)	03712013^2^	Lateral resistivity	Short‐normal resistivity	168‐2118	1953
Culp 1 (CULP)	03706169^2^	Lateral resistivity	None	50‐1563	1946

The API Number is a unique “American Petroleum Institute” identifier assigned to each individual oil and gas well drilled in the United States, excluding the state code, which is 04 in all California wells. NWIS refers to the U.S. Geological Survey National Water Information System.

^1^ Digital copies of geophysical logs are available from USGS GeoLog Locator (U.S. Geological Survey 2021); TDS data are available from NWIS (U.S. Geological Survey 2018).

^2^ Digital copies of the logs are available from California Department of Conservation.

The digitized ER data from the selected sites were collected by different methods at different times; a summary of the electrical logs is presented in Table 1, and data are available from the supporting data release (Terry et al. 2021). The available ER data from borehole geophysical logs at each site varied; historical information from the 1940s and 1950s were typically limited to lateral resistivity logs whereas modern logs from wells drilled since the 1970s had more logging information. Where multiple types of ER datasets from boreholes were available, we selected one of the ER types for use in simulation giving preference to modern long‐normal resistivity logs followed by lateral resistivity logs. Short‐normal resistivity logs were available for many sites but were not used because they are designed to read the resistivity of any mud invasion near the borehole and do not represent ambient formation properties. The borehole geophysical logs also possessed associated lithological descriptions that were used to filter out nonsandy intervals from the dataset used in the simulation. As we will discuss below, it is important to note that we include the ER logs (Y) as probabilistic, “soft” information (encoded as $I^{\text{soft}}$) and the uncertainty related to the logging method is inherently included in the simulation by use of all log types in the ER‐TDS calibration procedure, discussed in the following sections and is further explored in Supplemental Information included with this article.

**Figure 4:**
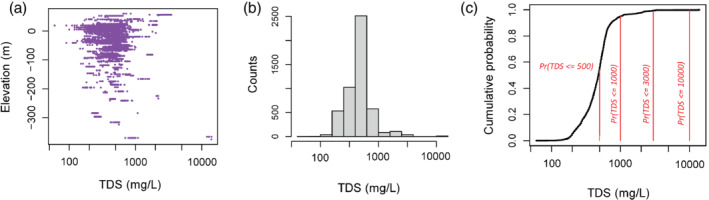
Summary of the TDS data: (a) all TDS sample data used in the study (Terry et al. 2021), plotted against NAVD88 elevation; plotting point is the center point of the screened interval, or the well depth when the screened interval was not recorded; (b) histogram of TDS values in the dataset; (c) cumulative probability of the full TDS dataset with cutoffs of interest (z) demarcated as red lines.

Water sample TDS data were available in the Montebello area from water‐supply or monitoring wells (Metzger et al. 2018). These data were primarily collected from the year 2000 or later (73% of the total); however, some TDS samples were collected as early as 1951. The TDS data were clipped to a 3‐mile (4.828 km) buffer zone surrounding the Montebello Oil Field administrative boundary (Figure 1b). The TDS measurements were recorded as latitude, longitude, and depths below land surface to the top and bottom of the well screened interval (where known) or bottom of the well where screened interval was unknown. Depths were converted to elevations relative to the NAVD88 elevation datum using the 1/3 arcsecond (∼10 m lateral resolution) USGS National Elevation Dataset (U.S. Geological Survey 2017) by selecting the nearest available point. A total of 4924 TDS measurements from 422 unique geospatial locations were used (Figures 1b, 2, 4) including compiled information from the U.S. Geological Survey (2018), the Water Replenishment District of Southern California (2019), the California State Water Resources Control Board (2018b), the California Department of Water Resources (2018), Metzger et al. (2018, 2020). These TDS data,Z, were lognormally distributed, ranged from 60 to 14,000 mg/L, and had a median value of 480 mg/L (Figure 4b). The global cumulative probabilities for data falling below each TDS threshold (z) are shown in Figure 4c.

The ER (Y) and TDS (Z) data ranged in elevation from −500 to +100 m relative to the NAVD88 datum; therefore, the study area was clipped to this interval.

### Geological Information and Water Table Elevation

Geological heterogeneity in the estimation area was evaluated. Chronostratigraphic layer information estimated from Ponti et al. (2014) consists of the elevation of several unconformities in the Montebello area observed from oil industry seismic reflection surveys and borehole log data. We used this information to set an additional constraint on the lower bound of our estimation grid based on the upper surface of the oil‐bearing Repetto Formation. We evaluated the possibility of directly incorporating all chronostratigraphic layers overlying the Repetto into our estimation procedure but lacked sufficient data to form a relation between the chronostratigraphic layers and ER or TDS.

Estimated water table elevation contours were available from Water Replenishment District of Southern California (2017) for most of the study area, ranged from −6 to 46 m elevation relative to the NAVD88 datum, and indicated an average groundwater flow direction from northeast to southwest. Water table elevation was not available in the most northeastern edge of the study area, and so a multivariate linear regression incorporating spatial position and land‐surface elevation was used to extend water table elevations into this region. Estimated water table elevation from this method ranged from −6 to 75 m elevation. This information was used to establish an upper bound to the study domain.

### Calibration Datasets

**Figure 5:**
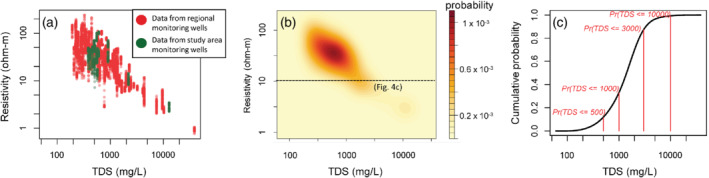
TDS and electrical resistivity (ER) calibration data set for boreholes in the Los Angeles Basin examined in this study: (a) TDS versus resistivity for regional monitoring wells shown in Figure 1a, (b) TDS‐resistivity kernel density estimated prior probabilities, and (c) normalized cumulative probability density for resistivity = 10 Ω‐m.

The calibration data described in this section were used to provide probability distributions used to encode soft data inputs for the simulations, in other words, to convert Y to $I^{\text{soft}}$. Co‐located borehole ER measurements and monitoring well TDS values were available from the larger Los Angeles Basin (Water Replenishment District of Southern California 2018; Figure 1a) as well as the COMM and MONT multiple groundwater well monitoring sites in the study area, but not at other locations within the main study area (the region shown in Figure 1b). The data from the Los Angeles Basin span the same chronostratigraphic layers present in the Montebello study area (Ponti et al. 2007, 2014). The screened interval of the monitoring wells (typically 6.1 m) was larger than the vertical sampling intervals of the ER measurements (0.3 m); therefore, TDS measurements were replicated for changing values of ER along the screened intervals. A TDS‐ER crossplot of the calibration dataset is shown in Figure 5a and generally shows the expected indirect correlation between ER and TDS, with a similar relationship observed between the regional data and simulation area data (red and green points in Figure 5a, respectively). A comparison to groundwater specific conductance (see Supporting information) indicated that the TDS data were linearly related to specific conductance (Figure S4a), and that specific conductance and ER generally follow an Archie relation (Archie 1942) with a best‐fit formation factor of 4.79 (*R* ^2^ = 0.91) (Figure S4b). The ER range from the calibration data (Figure 5a, approximately 0.75 to 244 Ω‐m) encompasses that of the ER of borehole geophysical logs from the simulation area (Figure 3, 2.77 to 159 Ω‐m). TDS values within this calibration dataset also cover the range of interest from less than 500 mg/L to greater than 10,000 mg/L.

### Assignment of Prior Probabilities

Using the calibration data in Figure 5a 2D kernel density estimators were developed in R (R Core Team 2021) using the kde2d function (Venables and Ripley 2002) for TDS‐ER data. The 2D kernel density estimate was used to provide a probability density estimate over a two‐dimensional grid using a Gaussian kernel, where 100 bins were used in each direction spanning the variable ranges. The bandwidth of the smoothing kernel, which effectively governs the smoothing between bins, was set to a quarter of the variable range. Probability densities were normalized by the area of each 2D bin and are presented as probabilities in Figures 5b. The visual density of data in Figure 5a is reflected as higher prior probabilities in Figure 5b.

**Figure 6:**
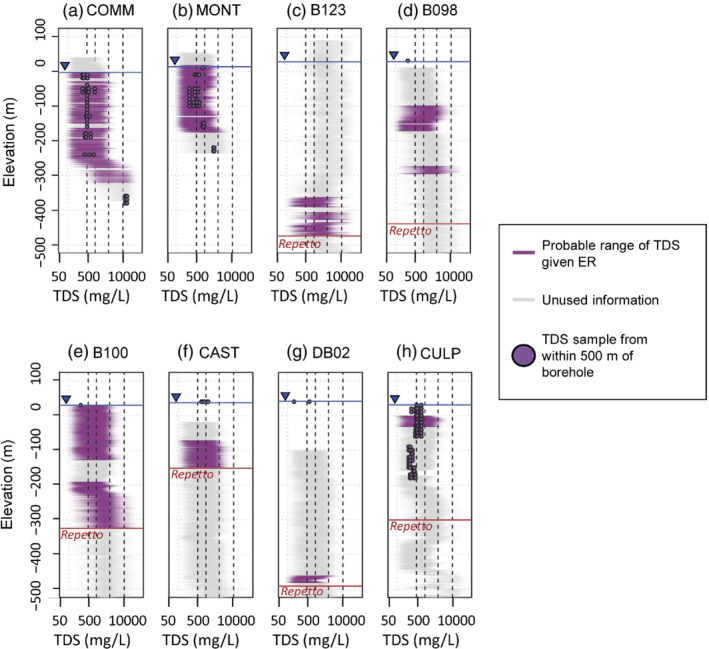
TDS probability given electrical resistivity (ER) log data, using the kernel density estimator shown in Figure 5b; TDS sample data from within 500 m of the boreholes are projected onto the plots for comparison; “unused information” refers to data above the water table, within the Repetto Formation, or corresponding to nonsandy lithological units.

The indicator coding of the soft data could now be accomplished using these probability distributions to turn Y into $I^{\text{soft}}$. An illustrative example is shown in Figure 5c for a hypothetical datapoint possessing a 10 Ω‐m ER. The dashed line in Figure 5b is shown as a 1D cumulative probability distribution for TDS in Figure 5c. The values of this curve at the three TDS cutoffs would be used as the indicator‐coded soft data prior probabilities (i.e., forming one row of $I^{\text{soft}}$). In this hypothetical case, prior information suggests that such a datapoint has approximately an 11% chance of having TDS of less than or equal to 500 mg/L, a 33% chance of having a TDS of less than or equal to 1000 mg/L, approximately 88% chance of having TDS less than or equal to 3000 mg/L, and nearly a 100% chance of having TDS less than or equal to 10,000 mg/L. Therefore, this datapoint would form a row of $I^{\text{soft}}$ and be coded as [0.11, 0.33, 0.88, 1.00] for input to the COSISIM indicator simulation framework. Figure 6 shows probable ranges of TDS for the ER information within the study area with nearby TDS data (within 500 m) projected on to the profiles. It can be seen in Figure 6 that local TDS values fall within probable TDS ranges predicted from the ER data. In fact, TDS data appear to fall within probable ranges predicted by ER data even outside of sandy lithologies, indicating that in future work it may be appropriate to include nonsandy lithologies within the estimation framework given the probabilistic nature of incorporating the ER data. For this study, however, we have limited inclusion of ER data to regions with a recorded sandy lithology.

The hardness parameters, B(z), used to infer covariance and cross‐covariance between hard and soft data are also derived from the calibration dataset. This analysis was performed in R (R Core Team, 2021) based on equations found in Deutsch and Journel 1998. The calculated values for the B(z) parameters were 0.229, 0.556, 0.803, and 0.454 for the z cutoffs (500, 1000, 3000, and 10,000 mg/L TDS, respectively). These values indicate the relative ability of ER data to successfully predict TDS for each class, with values closer to 1 equivalent to a stronger predictive ability. The lowest B(z) value is for the freshest salinity class (less than or equal to 500 mg/L TDS), and is reflected in Figure 5a by the wide spread of resistivity values measured for TDS in this range.

Soft and hard data were snapped to the nearest voxel center points within the simulation grid. In cases where multiple data points occupied the same voxel, prior probabilities for hard data falling below the specified thresholds within that voxel were computed based on relative counts of the data. In cases where multiple soft data fell into the same voxel, probabilities of each data point falling within a given voxel were computed based on the kernel densities established (Figure 5b), and the average probability distribution was used.

### Variograms

To estimate spatial correlation structure in the dataset, directional experimental semivariograms were computed based on the available data within the study area using combined indicator‐transformed TDS ($I^{\text{hard}}$) and ER data ($I^{\text{soft}}$) at the first z cutoff (TDS ≤ 500 mg/L), as this TDS value is the approximate median value in the dataset. In other words, the input data for experimental semivariograms was a concatenated vector of the first columns of $I^{\text{hard}}$ and $I^{\text{soft}}$, representing the probability of each of the datapoints being less than the median TDS value (500 mg/L). This approach is consistent with the median indicator cokriging method we adopted for simulation (see following section and previous section on covariance inference).

**Figure 7:**
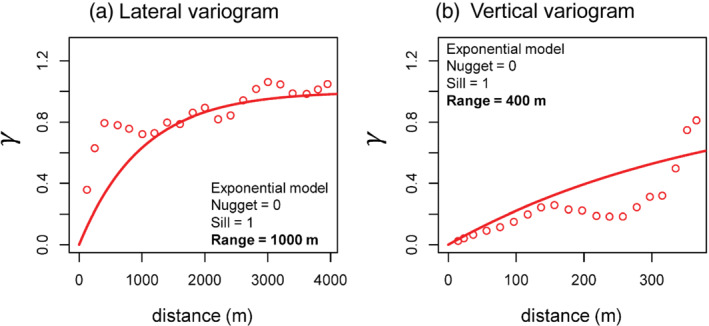
Directional indicator semivariograms used to estimate spatial correlation structure in the dataset using the GSLIB program GAMV(shown as hollow circles): (a) lateral (horizontal) variogram and (b) vertical variogram; lines indicate the variogram model fits. *γ* = standardized semivariance (unitless).

Experimental directional semivariograms were computed using the GSLIB program GAMV (Deutsch and Journel 1998; Figure 7, shown as hollow circles). The GAMV program allows the user to choose certain variogram parameters such as the total number of lags, the lag separation, and the lag tolerance (“overlap” between successive lags), as well as directional components for capturing point pairs such as the azimuth, azimuthal tolerance, horizontal bandwidth, dip angle, dip angle tolerance, and the vertical bandwidth. Please see GSLIB documentation (Deutsch and Journel 1998) for detailed descriptions and pictorial representations of these parameters. Parameter choices for computing the vertical and lateral semivariograms are shown in Table 2.

**Table 2:** Directional Experimental Semivariogram and Model Fit Parameters Used for the GSLIB Program GAMV

	Lateral	Vertical
Number of lags	20	50
Lag separation (xlag, m)	200	20
Lag tolerance (xltol, m)	200	20
Azimuth (azm, clockwise degrees from north)	0	0
Azimuthal tolerance (atol, degrees)	180	15
Horizontal bandwidth (bandh, m)	100,000 (infinite)	100
Dip angle (dip, degrees up from horizontal)	0	−90
Dip angle tolerance (dtol, degrees)	25	45
Vertical bandwidth (bandv, m)	200	100
Model	Exponential	Exponential
Nugget	0	0
Sill	1	1
Range (m)	1000	400

Experimental variogram parameters were chosen to estimate a lateral variogram with larger lag separations (200 m), compared to the vertical variogram (20 m) given the relative size of the study area (much wider than deep) and data density in these directions. The lateral variogram used a 180° azimuth and large horizontal bandwidth to ensure point pairs were captured in all horizontal directions, while a dip tolerance of 25° and vertical bandwidth of 200 m allowed for more flexibility in capturing distant point pairs. The vertical variogram used a dip angle of −90° to specify the downward direction, with a 45° dip tolerance and vertical bandwidth of 100 m to capture point‐pairs in relatively wide inverted “cones” for each lag. A slight azimuthal angle tolerance (15°) and horizontal bandwidth was also included. Sills were standardized to 1 in both lateral and vertical variogram calculations, as is appropriate for input to indicator simulation.

Experimental semivariograms were fit to variogram models using the R package gstat (Pebesma 2004; Gräler et al. 2016) and were slightly adjusted manually to ensure the same standardized sill (1) and nugget (0) for lateral and vertical directions; a necessary condition for input to COSISIM and generally supported by the experimental semivariograms (Figure 7). The final model fits are shown as solid curves in Figure 7. In both the lateral and vertical cases, exponential variogram models provided a reasonably good fit to experimental variograms and showed ranges of 1000 and 400 m, respectively; overall indicating a horizontally layered structure. The vertical variogram (Figure 7b) depicts a “hole effect” at larger separation distances (Journel and Huijbregts 1978) which may be an artifact produced by lithological layering or data sparsity in the vertical direction.

### Stochastic Conditional Indicator Simulation Approach

**Figure 8:**
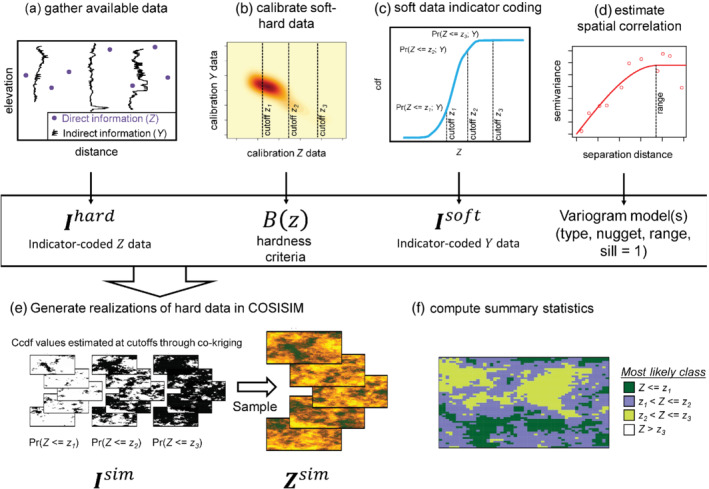
Conceptual diagram of the indicator simulation approach used in this study; (a) spatial direct (hard data, Z) and indirect (soft data, Y) data are gathered; (b) the indirect information is probabilistically related to direct data using a calibration dataset and the B(z) parameters are estimated; (c) direct and indirect information are coded as probabilities ($I^{\text{hard}}$ and $I^{\text{soft}}$) at each of the cutoffs, z; (d) a variogram model is estimated from variogram fitting to $I^{\text{hard}}$ and $I^{\text{soft}}$ data; (e) using the information from (a to d) conditional indicator simulation in COSISIM is performed to generate many equiprobable realizations, $Z^{\mathrm{est}}$, over the simulation grid and these results are output in the matrix $Z^{\mathrm{sim}}$; (f) summary statistics or other information are extracted from the rows of $Z^{\mathrm{sim}}$ for decision‐making or further modeling.

We used the SGeMS program COSISIM (Remy 2005) to perform conditional indicator simulation over a 3D grid. Our approach consists of the following general steps, which are conceptually illustrated in a 2D example Figure 8:

1. [1] Available data, consisting of direct measurements of TDS (Z) as well as borehole geophysical measurements of ER that indirectly inform TDS (Y), are gathered from the study area (Figure 8a).
2. Indirect information (Y) is calibrated to direct information (Z) as prior probabilities using a kernel density estimator (Figure 8b), and the hardness parameters (B(z)) that summarize the covariance between hard and soft indicator data are calculated.
3. For each of the k=4 TDS cutoffs (z), hard data are encoded into $I^{\text{hard}}$ as 1 if they are less than or equal to the given cutoff (0 otherwise); while a cumulative conditional distribution function based on the relationship formed in step 2 is used to assign conditional prior probabilities for the soft data for each of the cutoffs to form $I^{\text{soft}}$ (Figure 8c).
4. Spatial correlation structure of the study area is established through variogram fitting (Figure 8d) of the data in $I^{\text{hard}}$ and $I^{\text{soft}}$.
5. A simulation grid is established with $n_{\text{grid}}$ cells, and $n_{\mathrm{sim}}$ realizations are generated through indicator co‐kriging which honors the prior probabilities encoded into $I^{\text{hard}}$ and $I^{\text{soft}}$, hardness criteria (B(z)), and variogram parameters (nugget and range) that define the correlation structure (Figure 8e). Data values are drawn from the simulated ccdf values ($I^{\mathrm{sim}}$) for each realization and are output in terms of the original hard data (Z) units (i.e., mg/L for TDS) as a matrix of $n_{\text{grid}}$ grid cells by $n_{\mathrm{sim}}$ realizations, $Z^{\mathrm{sim}}$.
6. Summary information is gathered from the rows of simulated data in $Z^{\mathrm{sim}}$, which includes maps of the median simulated data and the most likely TDS category (Figure 8f).

Sequential indicator simulation in COSISIM was run with ordinary median indicator cokriging using the vertical and lateral variogram parameters (Figure 7), the specified z cutoff values, the B(z) hardness criteria, and the $I^{\text{hard}}$ and $I^{\text{soft}}$ data as input. COSISIM was used to generate m=500 realizations of log_10_(TDS) on a 3D grid in the area of the Montebello Oil Field within a subsurface region 16.4 × 12.3 km^2^ laterally, and 0.625 km thick vertically. Voxel sizes were 100 × 100 × 25 m^3^. Therefore there were$\;n_{\text{grid}}=504,300$ voxels in the simulation grid and the final estimates of log_10_(TDS) were output in $Z^{\mathrm{sim}}$; an $n_{\text{grid}}\times n_{\mathrm{sim}}$ matrix. Because COSISIM is limited to cuboid simulation grids, $Z^{\mathrm{sim}}$ results were clipped to the estimated variable base of water‐bearing geological units (top of the Repetto Formation) and the variable estimated groundwater elevation following simulation (retaining 57% of the simulated elements).

Probabilities of TDS falling into a particular class (less than or equal to 500, 500 to 1000, 1000 to 3000, 3000 to 10,000, greater than 10,000 mg/L) were computed by counting the number of times TDS values were simulated within each bin class, and then dividing by $n_{\mathrm{sim}}$. The most likely class for a given voxel was selected as the class with the highest probability for that voxel. Exceedance probabilities for a given TDS threshold were calculated based on the relative number of times values were simulated above the threshold.

## Results

### Results from Simulation

**Figure 9:**
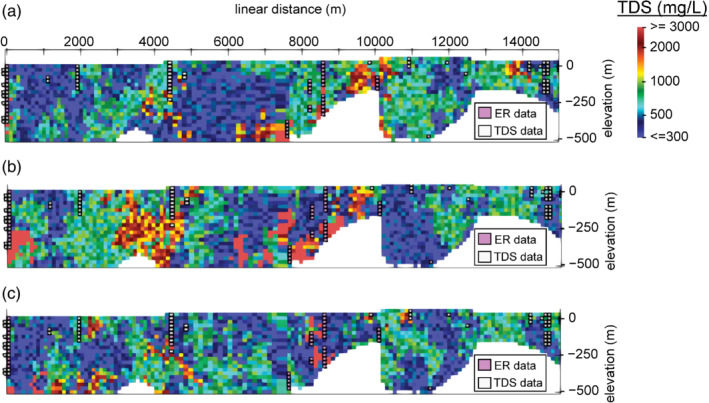
2D display of 3 of 500 example simulation results shown along the transect pictured in Figure 1b; locations of TDS data within 200 m of the profile are projected. ER = electrical resistivity.

**Figure 10:**
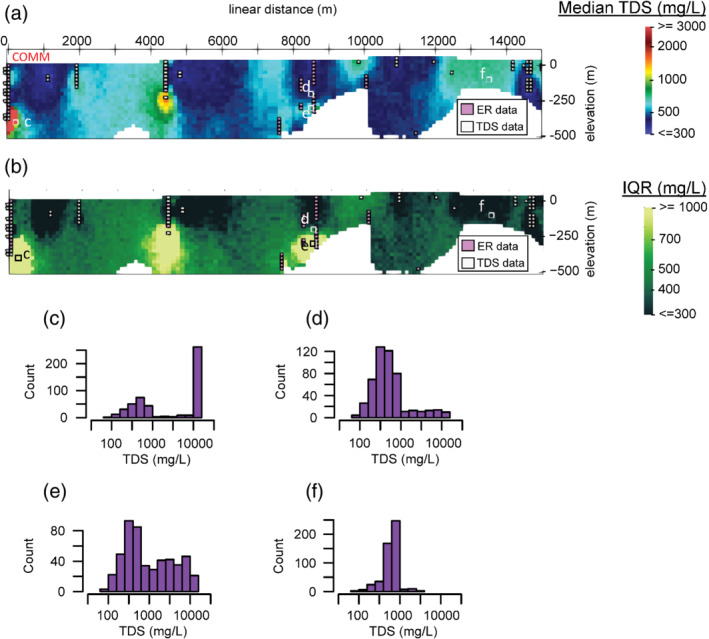
2D display of summarized results shown along the transect pictured in Figure 1b; (a) the median values from all 500 realizations; (b) interquartile range (IQR) of values simulated; (c to f) example distributions of simulated TDS values from locations labeled c to f in (a and b); Commerce‐1 well (COMM) is annotated in (a).

Figure 9 depicts 3 out of the 500 total simulation results along the 2D profile that intersects available ER data as shown in Figure 1b. Figure 10a shows the median of all indicator simulations along the same 2D transect, while Figure 10b shows the interquartile range. Histograms from all simulated values at four example voxels are shown in Figures 10c‐10f, with locations indicated on Figure 10a and 10b. These histograms demonstrate the ability of geostatistical simulation results to be viewed probabilistically. The histograms indicate the relative probability of TDS at each point resulting from the geostatistical model, subject to the conditions of the model. The relative number of simulated TDS values falling into each bin of the histogram can be converted to a relative probability for each bin and can therefore be used to approximate a posterior (i.e., after simulation) probability distribution. These posterior probability distributions may be fit to parametric models (e.g., a normal or exponential distribution) or be nonparametric (e.g., probability is explicitly defined for ranges of TDS values). More appropriate methods for estimating the expected value and uncertainty at voxels can be used depending on the form of the posterior distribution. Here, for example, given that the simulated TDS values appear not entirely normally distributed in log space, we opt to present median values as the metric to describe central tendency as opposed to the mean (Manikandan 2011). In the case of Figure 10c, a bimodal distribution is present, reflecting locally saline water (TDS greater than 10,000 mg/L) measured in the COMM well, which is surrounded by primarily fresh water (TDS less than or equal to 1000 mg/L, Figure 6a).

**Figure 11:**
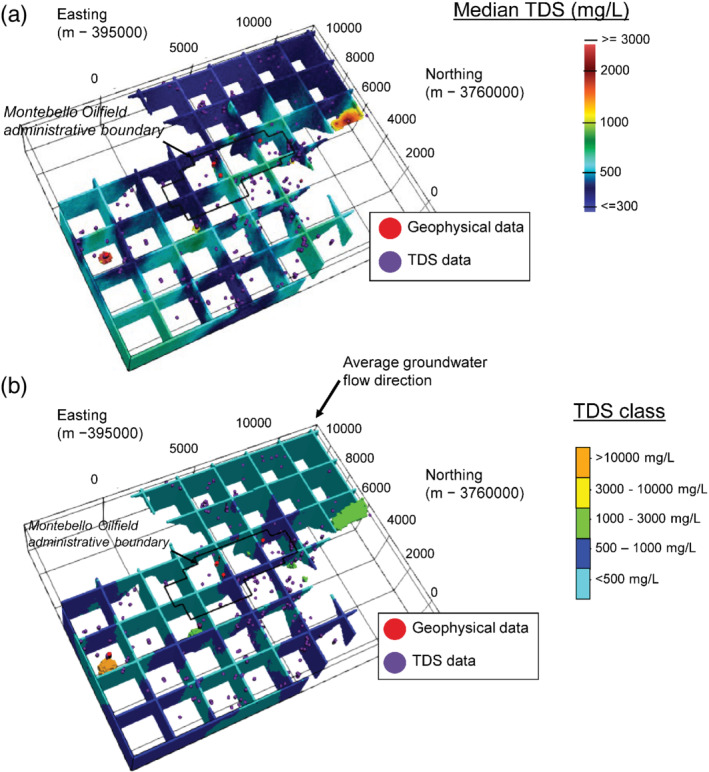
Summary statistics from COSISIM conditional indicator simulation shown as fence diagrams; (a) median TDS values; (b) most likely TDS class estimated with classes of TDS greater than 1000 mg/L highlighted where they appear.

Figure 11a depicts a fence diagram of the median simulated values throughout the study area, with TDS sample locations and ER data locations overlain and areas estimated as moderately to highly saline shown. Figure 11b depicts the spatial distribution of the most likely TDS class: less than or equal 500 mg/L and 500 to 1000 mg/L (fresh), less than or equal to 3000 mg/L but greater than 1000 mg/L (slightly saline), less than or equal to 10,000 mg/L but greater than 3000 mg/L (moderately saline), and greater than 10,000 mg/L (highly saline). The most likely class for each voxel was determined as the one containing the largest proportion of the simulated values. Though the majority (99%) of the study area consists of fresh water, there is an apparent increase in TDS within the range of freshwater in the direction of groundwater flow toward the southeast portion of the study area. Also, comprising a relatively small amount of the study area (less than 1% total), localized zones of slightly to highly saline groundwater were estimated. These areas indicate locally high TDS and/or low ER in the underlying data. The highly saline area associated with the COMM well in the southwest portion of the study area (Figure 6a) occurs at depth below −250 m elevation possibly reflecting deeper groundwater, whereas the moderately saline areas generally appear shallower (median elevation is −50 m) possibly reflecting more recent recharge.

**Figure 12:**
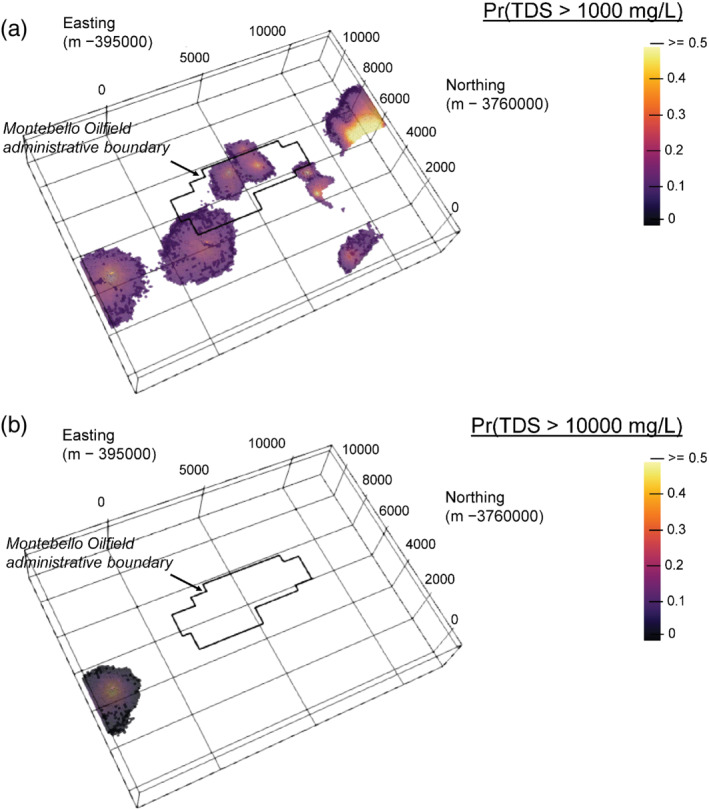
Exceedance probabilities from simulation; (a) areas with 10% or greater probability of exceeding 1000 mg/L TDS; (b) area with 10% or greater probability of exceeding 10,000 mg/L TDS.

Figures 12a and 12b highlight regions of the study area with at least 10% probability of exceeding the fresh (TDS = 1000 mg/L) and highly saline (TDS = 10,000 mg/L), respectively. With the exception of the area surrounding the COMM well, there are no other areas with greater than 10% chance of falling in the highly saline range. However, additional areas are revealed that may be at least slightly saline (greater than 10% probability) in Figure 12a, particularly within the Montebello Oil Field Administrative area. Predictions in this area are influenced by a higher density of ER logs (B123, B098, B100), which exhibit prior probability distributions in the saline range (Figures 6c‐6e). The influence of TDS data within the correlation range and uncertainty in the ER‐TDS relationship, however, results in the assessment on these areas being most likely fresh. Nevertheless, a useful feature of the simulation approach is the ability to define such probabilistic thresholds to evaluate relative risk and/or prioritize areas for additional data collection.

### Model Validation

To assess the ability of simulation results to correctly predict salinity, a 4‐fold cross‐validation (resampling without replacement) approach was used. In 4‐fold cross‐validation, the TDS dataset is divided into 4 “folds” or bins, which are used as test datasets for simulation results trained on the remaining data. In other words, four new batches of 500 COSISIM simulations were run, each with 25% of the TDS (hard) information withheld to compare the accuracy of predictions. In this case, “accuracy” was assessed by the ability of the model to estimate the most likely class of datapoints that were left out of each cross‐validation run; leaving out a few datapoints with equiprobable classes. Table 3 presents a summary from each of the 4 folds. The number of incorrectly classified points ranged from 8% to 19% of the test datasets. In the first two cross‐validation folds, 1 to 3 points were incorrectly estimated as most likely falling into the less than 500 mg/L class, when the true most likely class for those points was moderately saline (1000 to 3000 mg/L).

**Table 3:** Results from Fourfold Model Cross‐validation of the Total Dissolved Solids Dataset

	Fold 1	Fold 2	Fold 3	Fold 4
Incorrect/number of data in test set	16/181	27/182	14/184	34/183
% test set incorrectly classified	9%	15%	8%	19%
Cases where prediction was off by more than 1 class	Three moderately saline points were classified as fresh (≤500 mg/L TDS)	One moderately saline point was classified as fresh (≤500 mg/L TDS)	None	None

Overall, in cases of misclassification, the model tended to underpredict TDS, which is not surprising given the global cumulative distribution function for TDS (Figure 4c) indicates a global probability of 0.95 for freshwater (TDS less than or equal to 1000 mg/L) based on all available data in the study area. In areas outside of the correlation range of datapoints, it is therefore extremely likely datapoints will be simulated as fresh given no other information.

## Discussion

The sequential indicator simulation approach used in this paper allows for integration of multiple data types toward a common estimation goal (here, to estimate groundwater salinity). Direct information on TDS is limited in the Montebello Oil Field. Although indirect ER data are available, there is uncertainty in the relationship between TDS and ER, the latter of which is sensitive to other factors including porosity, lithology, degree of compaction, and temperature (Jorgensen 1996). Petrophysical and empirical relationships linking ER and TDS exist (e.g., Alger and Harrison 1989; Hamlin and de la Rocha 2015), but there is uncertainty in this relationship given the likely variation in physical properties in space. One possible method for capturing this uncertainty is to estimate petrophysical properties as part of the TDS estimation framework itself (e.g., Stephens et al. 2019). Here, however, we treat geophysical data as soft information (Y), coded as probabilities ($I^{\text{soft}}$) based on a calibration with hard (TDS) data (Z), which offers a means of dealing with this uncertainty in the estimation procedure.

A strength of the sequential indicator simulation approach is a robust ability to capture estimation uncertainty, similar to other Bayesian methods (e.g., Blatter et al. 2019). Histograms of simulated values can be used to estimate probability distributions, which offer detailed insight into TDS likelihood and uncertainty throughout the study area and need not be limited to summary statistics derived from normal distributions (e.g., the mean and standard deviation). Posterior probability distributions can be used as prior distributions in further stochastic modeling (Thijssen and Wessels 2020), such as stochastic groundwater modeling or additional salinity modeling in the Montebello area.

There are, however, several limitations to the approach and results presented in this work. First is that we must assume global covariance structure, which is based on variograms that use relatively sparse data (Figure 7), particularly in the vertical direction. The range of the lateral and vertical variograms exert relatively strong control over the results of the geostatistical simulations. Significant effort was made to fit variogram models that realistically reflect the correlation observed in the data; however, without additional information we cannot verify the robustness of these models and there is no consensus on an objective means for fitting variogram models (Chiles and Delfiner 2012).

A second assumption in this modeling is that ER and TDS data were collected simultaneously and represent a single slice in time, when in fact the data used span many years. Data from some borehole geophysical logs were collected in the 1940s (for example, Baldwin 100 was logged in 1943, Table 1), while some TDS samples were collected in the 2010s. The data locations for TDS were also subject to uncertainty, as the screened interval of wells was not always known. Furthermore, it is assumed that all TDS data are comparable despite both known and unknown variation in the analytical method for measuring TDS, though such variability is expected to be slight (see Supporting information). We capture uncertainty in data locations and analytical method by treating input data probabilistically; resampling information to the resolution of the simulation grid and using this information to form a prior probability distribution function (based on counts of data falling into the different TDS classes within that pixel). Similarly, ER data undergo this same resampling procedure and are also subject to uncertainty assessed when forming the kernel density estimator (Figure 5b). Nevertheless, future work will likely investigate temporal components of the likely TDS distribution in the study area.

A third assumption is that ER data from borehole geophysical logs collected using various instrumentation during different periods in time (see Supporting information), with different inherent measurement volumes, can be combined to yield an internally consistent ER data set that is appropriate to assign probabilities from ER vs. TDS data from Los Angeles Basin groundwater monitoring wells. However, at least for the monitoring well ER data, geophysical logging methods were reasonably consistent (see Supporting information). Furthermore, by coding the indirect ER information as probabilities based on actual variability observed using different logging methods (see supplemental information), such uncertainty is captured in the estimation approach.

Finally, though we have attempted to maximize use of all available data in our approach, areas of the model remain data‐sparse or completely uninformed (e.g., outside of the correlation range of data points). Such sparse areas manifest as elliptical structures in resulting salinity models (Figures 11, 12) as they may be controlled by only a few data points and the lateral/vertical correlation range. However, these results are useful for identifying priority regions for closer investigation, further data collection (monitoring wells) or digitization of historical borehole geophysical logging data that could better inform the approach.

Despite these assumptions, we maintain that the geostatistical simulation approach used here is well suited to the problem, given that the outcomes are presented as probabilistically and not deterministically, and are readily updated with the addition of new data. Given the ongoing interest and importance of salinity modeling in the Montebello area, the future will yield many new sources of information. For example, although some borehole geophysical logs from the area have been digitized, there are many more that have yet to be made available. New monitoring wells, and additional data from existing monitoring wells, will provide additional information. Finally, supporting geophysical data (for example, time‐domain electromagnetics) have the potential to provide ER information at relevant spatial scales and depths for salinity mapping (Delsman et al. 2018; Ball et al. 2020; Lane et al. 2020). Alternatively, results from our salinity model could form the priors for a Bayesian inversion of airborne electromagnetic geophysical data (e.g., Blatter et al. 2018).

## Conclusions

Geostatistical modeling indicated that the aquifer above the Montebello Oil Field is typically fresh (less than 1000 mg/L TDS) with a trend of increasing salinity in the direction of average groundwater flow toward the southwest. Isolated zones of slightly saline (1000 to 3000 mg/L TDS) were predicted, though these zones were typically shallow and may correspond to recent return flows. Though not deemed the most likely scenario, deeper areas of slightly saline groundwater were also predicted with at least 10% probability throughout the study area, particularly within the Montebello Oil Field administrative area. An isolated zone of deep (below −250 m elevation) high salinity groundwater was estimated to occur in the southwest corner of the study area; informed by direct TDS measurements from a local well and low ER measured in this area.

Environmental management decisions often must be made despite limited data availability and uncertainty. Therefore, decision frameworks incorporating a variety of information into a single estimation approach that honors and propagates this uncertainty are valuable. The geostatistical simulation approach demonstrated in this paper provides a convenient means for combining sparse direct information on salinity and more widely distributed yet indirect information to produce probabilistic estimates that can be used to guide decision making. Although there is uncertainty in terms of geological heterogeneity, assumptions made about the data correlation structure, and lack of information in certain areas of the estimation region, these uncertainties may be reduced with the collection of additional data. This work provides a proof‐of‐concept demonstration for expanded analysis over other areas of the aquifer with additional sources of information, or for modeling salinity changes over time where time‐lapse data available. The approach used in this work is readily adapted to such tasks.

## Authors' Note

The authors do not have any conflicts of interest or financial disclosures to report.

## Supporting information


**Appendix S1.** Additional analysis to support modeling assumptions, including the appropriateness of using different types of borehole electrical geophysical logs and the validity of using data to calibrate the model from outside the model domain. In addition, a more detailed description of the parameters used in the Markov‐Bayes model is given. U.S. Geological Survey information products including supporting information are peer reviewed and approved for publication consistent with Fundamental Science Practices policies ( https://pubs.usgs.gov/circ/1367/). Additionally, a supporting U.S. Geological Survey data release (Terry et al., 2021) contains all data and results used in this analysis.

## 1

The following are the symbols used in this paper and a brief description:

Z: vector of hard data (TDS values in mg/L), length is the number of hard data, $n_{\text{hard}.}$
Y: vector of soft data (ER values in ohm‐m), length is number of soft data, $n_{\text{soft}}$
k: integer number of cutoffs for indicator coding (k=4 in this paper)
z: vector of cutoff values for indicator coding, length = k ($z_{1}=$500, $z_{2}=$ 1000, $z_{3}=$3000, and $z_{4}=$ 10,000 mg/L)
B(z): vector of hardness parameters, describing correlation strength between soft and hard data at each of the cutoff and used to circumvent need for explicit covariance and cross‐covariance estimates from the hard data, length = k
$I^{\text{hard}}$: matrix of indicator coded hard data, probability of hard data (Z) being less than or equal to each of the cutoff values (z), dimensions of $n_{\text{hard}}\times k$
$I^{\text{soft}}$: matrix of indicator coded soft data, probability of soft (*Y*) data being less than or equal to each of the cutoff values (*z*), dimensions of $n_{\text{soft}}\times k$
$n_{\text{grid}}$: integer number of spatial grid cells used in simulations
$n_{\mathrm{sim}}$: integer number of realizations generated from COSISIM
$I^{\mathrm{est}}$: matrix of indicator‐coded results from a single conditional indicator COSISIM realization, dimensions of $n_{\text{grid}}\times k$
$Z^{\mathrm{est}}$: vector of results from a single COSIM realization in original data units (log_10_[mg/L]) taken from sampling the interpolated/extrapolated conditional cumulative distributions in $I^{\mathrm{est}}$, length is $n_{\text{grid}}$
$Z^{\mathrm{sim}}$: matrix of results in original data units (log_10_[mg/L]) from all conditional indicator simulations, dimensions of $n_{\text{grid}}\times n_{\mathrm{sim}}$
