# Supplementary material for: Geostatistical Mapping of Salinity Conditioned on Borehole Logs, Montebello Oil Field, California
Source: Ground Water. 2021 Dec 16;60(2):242–61. doi: 10.1111/gwat.13155 (PMC9299866; doi:10.1111/gwat.13155)
Supplement: Supplementary file 1 — Appendix S1. Additional analysis to support modeling assumptions, including the appropriateness of using different types of borehole electrical geophysical logs and the validity of using data to calibrate the model from outside the model domain. In addition, a more detailed description of the parameters used in the Markov‐Bayes model is given. U.S. Geological Survey information products including supporting information are peer reviewed and approved for publication consistent with Fundamental Science Practices policies ( https://pubs.usgs.gov/circ/1367/). Additionally, a supporting U.S. Geological Survey data release (Terry et al., 2021) contains all data and results used in this analysis. [file GWAT-60-242-s001.docx]

Supporting information for manuscript: *Geostatistical mapping of salinity conditioned on borehole logs, Montebello Oil Field, California*

Authors: Neil Terry, Frederick D. Day-Lewis, Matthew K. Landon, Michael T. Land, Jennifer Stanton, and John W. Lane Jr.

# Appendix S1: Further analysis of assumptions made in the simulation approach

We were concerned that certain assumptions made in this study may render data inappropriate for use in constructing the geophysical simulation framework. Specifically, our three main concerns were:

[1] Different geophysical log types may not be comparable.

[2] Relationships derived from electrical resistivity (ER) and total dissolved solids (TDS) information outside of the simulation area may not be transferrable to the simulation area.

[3] Differences in analytical method for TDS data may affect results.

Therefore, we performed more detailed analysis to provide reassurance that these assumptions were reasonable.

## [1] Use of different geophysical logs

Available electrical geophysical logs varied, from lateral resistivity logs collected from oil wells as early as the 1940s to long-normal resistivity logs collected in monitoring wells from the 2000s. Table 1 presents a summary of the logs from the simulation area available at the time the study began and used in the analysis. As mentioned in the manuscript, our preference was to use modern long-normal (LN) resistivity logs, followed by lateral (LAT) logs. We deemed short-normal (SN) logs as inappropriate these may be affected by drilling mud and are not reflective of the surrounding formation.

Figure S1 shows corrgrams of data from the different log types from the regional wells (Figure S1). Pearson’s correlation coefficients are indicated on the corrgrams. Data from all logs were strongly positively correlated, with the strongest relationship observed between LN and SN data (correlation = 0.95), a 0.82 correlation between LN and LAT logs, and the weakest relationship was observed in the regional dataset between LN and IND logs (correlation = 0.79). Given the strong correlation between LAT and LN logs, these results provide confidence that including different geophysical ER logging methods in the simulation is reasonable.

**Figure S1** – corrgrams for data from different electrical geophysical log types from the regional monitoring wells. LN = long-normal resistivity, IND = induction resistivity, LAT = lateral resistivity, SN = short-normal resistivity.

## [2] Suitability of data from outside the simulation region

Electrical resistivity log data from 23 regional wells were available and used as calibration data in this study. These included two wells from within the study area (COMM and MONT). A plot of the different logs available are shown in Figure S2. Our primary concern was that the regional monitoring wells may not encompass the range of resistivity and subsurface conditions as those from within the study area. The boxplot in Figure S3 was generated in response to this concern, which shows the distribution of ER values from simulation area logs, the MONT logs, the COMM logs, and the regional study area. It can be seen that the regional ER values completely encompass the MONT, COMM, and simulation area log values. Furthermore, the bulk of the values for the regional logs and simulation area logs fall within a similar range.

However, despite falling within a similar range of values, there was still concern that lithological variations between the regional wells and conditions in the study area may differ, thus affecting TDS-ER relationships developed in the calibration. Therefore, though we do not explicitly employ an Archie-like (Archie, 1942) relationship in the manuscript, we evaluated what such a relationship would look like given our available data.

Collocated groundwater specific conductance (SpC), TDS, and ER data were available from the regional data (including the MONT and COMM locations). As expected, a very strong relationship (R^2^ = 0.99) between SpC and TDS was observed (slope = 0.65, Figure S4a).

In an Archie formulation under 100% water saturated sands, the relationship between bulk resistivity (i.e., measurements of both the formation and the pore fluid) and SpC can be related by a formation factor – a term that collectively accounts for the effect of the structure of the formation on the bulk resistivity (porosity, interconnectedness of the pores, etc). We compare SpC with ER (plotted as bulk conductivity = 1/ER) in Figure S4b and fit a linear model to estimate a formation factor (the slope of this model), which was estimated at 4.79 (R^2^ = 0.91). This value is similar to the lower range of those estimated by Stephens et al. (2018), and is within the range of values measured for clean sands (Garba et al. 2019). Though the data clearly fit to an Archie-like relationship, variability observed may be due to effects such as temperature, varying lithology (and porosity), or the logging type (all log types were combined for this analysis). Though we do not have detailed lithological or temperature information from the study area, by including ER data from this dataset in a probabilistic sense we effectively incorporate a related degree of uncertainty from our lack of knowledge regarding varying lithological and temperature conditions.

As we expect ER to be linearly related to SpC, and SpC to be linearly related to TDS, we can develop a linear model that directly links ER to TDS. This relationship is shown in Figure S5, with the best linear model fit shown as a red line and linear fits bounding the data as dotted red lines. Note however that we do not explicitly calculate TDS from ER in the simulation approach, and this relationship is developed for demonstration only.

**Figure S2** – a comparison of the electrical logs available at regional monitoring wells. The positions of the wells are shown in Figure 1a of the manuscript.

**Figure S3** – Boxplots of the resistivity values from the simulation area (SA), the MONT combined electrical resistivity-total dissolved solids (ER-TDS) well (located within the simulation area), the COMM combined ER-TDS well (located within the general Montebello area), and the regional logs. In this figure, lines indicate the data median, filled colored boxes show the interquartile range, bars show the 1^st^ and 3^rd^ quartile -/+ the interquartile range multiplied by 1.5, and hollow circles are outliers.

**Figure S4** – (a) comparison of specific conductance with total dissolved solids (TDS); solid red line shows linear model fit; (b) comparison of bulk conductivity with groundwater specific conductance (SpC) (at 25 °C); solid red line shows linear model fit (slope = formation factor).

**Figure S5** – relationship between resistivity at the regional monitoring wells and salinity. Lines shown include the best least squares fit (solid, red) and lines that bound the data values encountered (dotted, red). Equations for each line are also shown.

## [3] Comparison of TDS measurement method

Methods for measuring TDS varied and was often unknown. A comparison of USGS-derived data from parameter codes 70300 (dissolved solids dried at 180 degrees Celsius, water, filtered, milligrams per liter), and 70301 (dissolved solids, water, filtered, sum of constituents, milligrams per liter) was performed for 112 data used in the Montebello study area to evaluate whether the differences in analytical method affected how TDS information would be classified in the geostatistical modeling approach used in this study. The mean and standard deviation of the absolute percentage difference between TDS values measured by each method was 4% and 3%, respectively. In 11/112 of the cases, a discrepancy in reported values from the two analytical methods occurred resulting in a “very fresh” (<= 500 mg/L) versus “fresh” (<=1000) mg/L categorical discrepancy. However, such discrepancies did not occur for any of the other salinity categories (“slightly saline”, “moderately saline”, “highly saline”).

**Appendix S2**: Discussion of parameters used in the Markov-Bayes model.

The hardness criterion, *B(z)*, describes how well soft data can be used to predict hard data classes with *B(z)* = 1 equating to a perfect prediction, and *B(z)* = 0 indicating no ability to use soft data to inform hard data for a class.

$$B\left( z \right)=m^{\left( 1 \right)}\left( z \right)-m^{\left( 0 \right)}\left( z \right)$$

$$m^{\left( 1 \right)}\left( z \right)=E\left\{ Y\left( z \right)|I\left( z \right)=1 \right\}$$

$$m^{\left( 0 \right)}\left( z \right)=E\left\{ Y\left( z \right)|I\left( z \right)=0 \right\}$$

$m^{\left( 1 \right)}\left( z \right)$ is a metric for describing the expected probability in the soft data, *Y*, for cases where hard data, *I*, has been coded as 1. So for example, in the ideal case where soft data perfectly predict the hard data, $m^{\left( 1 \right)}\left( z \right)=1$ in a 1:1 direct relationship, or $m^{\left( 1 \right)}\left( z \right)=0$ in a 1:1 indirect relationship. If there is no predictive power $m^{\left( 1 \right)}\left( z \right)=0.5$, given that the expectation is that half the time soft data can predict hard data, and half the time it cannot.

Conversely, $m^{\left( 0 \right)}\left( z \right)$ considers the expected value of soft data for cases where hard data has been coded as 0, and along similar lines, values of 1 or 0 indicate perfect predictive power, whereas a value of 0.5 indicates no predictive power. It can be seen then that in cases where $m^{\left( 0 \right)}\left( z \right)$ and $m^{\left( 1 \right)}\left( z \right)$ take on similar values, *B(z)* will be closer to zero, since there is little ability in the soft data to differentiate between cases where $I\left( z \right)$ is coded as 1 or 0.

Calculation of the $B\left( z \right)$ values is performed as follows. For each $z$ cutoff, calibration data falling within the categories of $I\left( z \right)=0$ and $I\left( z \right)=1$ are separated. The expected value is then calculated from the distribution of $Y\left( z \right)$ values (which are prior probabilities) from each group to compute $m^{\left( 0 \right)}\left( z \right)$ and $m^{\left( 1 \right)}\left( z \right)$.
